# Prion disease diagnosis using subject-specific imaging biomarkers within a multi-kernel Gaussian process

**DOI:** 10.1016/j.nicl.2019.102051

**Published:** 2019-10-25

**Authors:** Liane S. Canas, Carole H. Sudre, Enrico De Vita, Akin Nihat, Tze How Mok, Catherine F. Slattery, Ross W. Paterson, Alexander J.M. Foulkes, Harpreet Hyare, M. Jorge Cardoso, John Thornton, Jonathan M. Schott, Frederik Barkhof, John Collinge, Sébastien Ourselin, Simon Mead, Marc Modat

**Affiliations:** a Department of Medical Physics and Biomedical Engineering, University College London, London, United Kingdom; b School of Biomedical Engineering & Imaging Sciences, King’s College London, King’s Health Partners, St Thomas’ Hospital, London, SE1 7EH, United Kingdom; c Institute of Neurology, University College London, United Kingdom; d MRC Prion Unit at UCL, UCL Institute of Prion Diseases, London, United Kingdom; e NHS National Prion Clinic, University College London Hospitals NHS Foundation Trust, London, United Kingdom; f Dementia Research Centre, UCL Institute of Neurology, 8-11 Queen Square, London, WC1N 3BG, UK; g Amsterdam UMC, Vrije Universiteit, Amsterdam, the Netherlands

**Keywords:** Prion diseases, Inherited Creutzfeldt–Jakob disease, Sporadic Creutzfeldt–Jakob disease, Diagnosis, Biomarkers, Subjects’ stratification, Gaussian process

## Abstract

Prion diseases are a group of rare neurodegenerative conditions characterised by a high rate of progression and highly heterogeneous phenotypes. Whilst the most common form of prion disease occurs sporadically (sporadic Creutzfeldt–Jakob disease, sCJD), other forms are caused by prion protein gene mutations, or exposure to prions in the diet or by medical procedures, such us surgeries. To date, there are no accurate quantitative imaging biomarkers that can be used to predict the future clinical diagnosis of a healthy subject, or to quantify the progression of symptoms over time. Besides, CJD is commonly mistaken for other forms of dementia. Due to the heterogeneity of phenotypes and the lack of a consistent geometrical pattern of disease progression, the approaches used to study other types of neurodegenerative diseases are not satisfactory to capture the progression of human form of prion disease. In this paper, using a tailored framework, we aim to classify and stratify patients with prion disease, according to the severity of their illness. The framework is initialised with the extraction of subject-specific imaging biomarkers. The extracted biomakers are then combined with genetic and demographic information within a Gaussian Process classifier, used to calculate the probability of a subject to be diagnosed with prion disease in the next year. We evaluate the effectiveness of the proposed method in a cohort of patients with inherited and sporadic forms of prion disease. The model has shown to be effective in the prediction of both inherited CJD (92% of accuracy) and sporadic CJD (95% of accuracy). However the model has shown to be less effective when used to stratify the different stages of the disease, in which the average accuracy is 85%, whilst the recall is 59%. Finally, our framework was extended as a differential diagnosis tool to identify both forms of CJD among another neurodegenerative disease. In summary we have developed a novel method for prion disease diagnosis and prediction of clinical onset using multiple sources of features, which may have use in other disorders with heterogeneous imaging features.

## Highlights

• Subject-specific imaging biomarkers can be used to characterize Prion disease.
• Gaussian process can be used for the staging of prion disease patients.
• Probabilistic diagnosis are indicative of the patients symptoms progression.
• Gaussian process are effective for differential diagnosis.

## 1 Introduction

Prion diseases, also known as transmissible spongiform encephalopathies (TSEs), are a group of progressive neurodegenerative conditions, which cause cognitive impairment and neurological deficits (Collinge, 2001). All prion diseases involve a change in conformation of the normal cell surface prion protein (PrP) into multichain assemblies of abnormally folded forms.

Prion diseases can present with a wide spectrum of phenotypes, for several reasons, including importantly, variation of the coding sequence of the prion protein gene (*PRNP*) and the propagation of prion strains. Heterogeneity manifests in disease duration and clinical onset, symptomatology and the distribution of brain lesions, including spongiosis, neuronal loss, gliosis, reactive astrocytosis and deposition of misfolded prion protein (Parchi, Cescatti, Notari, Schulz-schaeffer, Capellari, Giese, Zou, Kretzschmar, Ghetti, Brown, 2010, Pocchiari, Puopolo, Croes, Budka, Gelpi, Collins, Lewis, Sutcliffe, Guilivi, Delasnerie-Laupretre, Brandel, Alperovitch, Zerr, Poser, Kretzschmar, Ladogana, Rietvald, Mitrova, Martinez-Martin, De Pedro-Cuesta, Glatzel, Aguzzi, Cooper, Mackenzie, Van Duijn, Will, 2004, Siddique, Hyare, Wroe, Webb, MacFarlane, Rudge, Collinge, Powell, Brandner, So, Walker, Mead, Yousry, Thornton, 2010).

The different forms of prion disease can be grouped by aetiology, accordingly, whether they are sporadic (unknown cause), inherited, or acquired (transmitted between mammals or humans). The sporadic form (sCJD) is the most common and it accounts for about 85% of the annual incidence of human prion disease. This form of CJD shows a significant neuronal loss, and vacuolisation within cell bodies and dendrites that gives a spongiform appearance to the cerebral cortex and deep nuclei (Johnson, 2005). sCJD is also characterised by the rapid progression of symptoms with prominent cognitive decline. The median time of survival after clinical onset is only 5 months, and 90% of the patients die within one year (Pocchiari et al., 2004). The inherited prion diseases (IPD) are caused by autosomal dominant inheritance of mutations in the *PRNP* gene, which in total are responsible for 10–15% of the incidence of human prion disease (Mead, 2006). Over thirty different mutations in *PRNP* have been found in patients presenting IPD, with about 95% of familial cases caused by four mutations (point mutations in codons 102, 178, 200 and 210) and insertions of five or six octapeptide repeats (Johnson, 2005). IPD has often an earlier clinical onset when compared with sCJD, which also presents a wide range of clinical onset ages, from 20 to 70 years old. The clinical course of IPD can be much longer than sCJD, up to 20 years (Mead, 2006). Due to these long clinical durations, IPD has a similar prevalence in the population than sCJD, despite a much lower incidence.

The clinical diagnosis of both forms of CJD can be challenging during life, due to the heterogeneity of the observed phenotypes, particularly in the earlier stages of the disease as they can mimic other neurodegenerative diseases. While the definitive diagnosis is still only possible by brain biopsy, the improved understanding of the pathogenesis of prion diseases have allowed definition of recognizable clinical features and a replicable diagnostic criteria in vivo (Fragoso et al., 2017). The diagnosis criteria are based on a set of neurological, cognitive and psychiatric observations (Mead, Ranopa, Gopalakrishnan, Thompson, Rudge, Wroe, Kennedy, Hudson, MacKay, Darbyshire, Collinge, Walker, 2011, Thompson, Lowe, Fox, Lukic, Porter, Ford, Gorham, Gopalakrishnan, Rudge, Walker, Collinge, Mead, 2013). Moreover, noninvasive imaging techniques, such as magnetic resonance (MR), computed tomography (CT), positron emission tomography (PET) or single-photon emission computed tomography (SPECT) have also been used to diagnose CJD. The qualitative assessment of neuroimaging data has proven to be useful to identify and characterise CJD among other pathologies  (Caobelli, Cobelli, Pizzocaro, Pavia, Magnaldi, Guerra, 2014, Manix, Kalakoti, Henry, Thakur, Menger, Guthikonda, Nanda, 2015, Schroter, Zerr, Henkel, Tschampa, Finkenstaedt, Poser, 2000, Zerr, Poser, 2002).

Several quantitative imaging measures and potential imaging biomarkers have been studied in order to improve the sensitivity and specificity of CJD diagnosis based on neuroimaging data. Siddique et al. (2010) investigated the cross-sectional, longitudinal and *post-mortem* cerebral magnetisation transfer ratios (MTR) as a surrogate for CJD progression. Highly significant associations were found between whole brain MTR and prion disease. Alner et al. (2012), explored the potential of using the cortical thickness as a biomarker in order to characterise IPD, especially the 6-OPRI and P102L variants. They showed significant differences in the mean cortical thickness between 6-OPRI patients and controls in temporal, cingulate, frontal, parietal and occipital lobes; whereas only the mean cortical thickness of the parietal lobe was relevant to distinguish controls from P102L patients. In a different study using MTR (Vita et al., 2015), differences between controls and symptomatic subjects were seen in the caudate, hippocampus, putamen and cortex. The brain progressive structural changes were also identified by applying longitudinal voxel-based morphometry (VBM): significantly greater rates of grey matter decline were observed, predominantly in the pons, the corpus callosum, the thalamus and the putamen, when comparing controls and symptomatic subjects (De Vita et al., 2017). Many studies have also explored the potential of fluid-attenuated inversion recovery imaging (FLAIR) in the detection of CJD (Collie, 2001, Kallenberg, Schulz-Schaeffer, Jastrow, Poser, Meissner, Tschampa, Zerr, Knauth, 2006, Murata, Shiga, Higano, Takahashi, Mugikura, 2002, Young, Geschwind, Fischbein, Martindale, Henry, Liu, Lu, Wong, Liu, Miller, Dillon, 2005). FLAIR has been shown to be reliable to detect the earlier stages of the disease than T2-weighted (T2w) and diffusion-weighted imaging (DWI). However, FLAIR is less effective than DWI to detect lesions, which become less prominent during the course of the disease (Shiga, Miyazawa, Sato, Fukushima, Shibuya, Sato, Konno, Doh-ura, Mugikura, Tamura, Higano, Takahashi, Itoyama, 2004, Young, Geschwind, Fischbein, Martindale, Henry, Liu, Lu, Wong, Liu, Miller, Dillon, 2005). The mean apparent diffusion coefficient (ADC) has proven to be a sensitive imaging biomarker for diagnosis based on abnormalities in the caudate, putamen and pulvinar nuclei. Research suggests that brain volume loss in inherited prion diseases is followed by cerebral ADC increase, and correlates with disease severity (Hyare, Thornton, Stevens, Mead, Rudge, Collinge, Yousry, Jäger, 2010, Hyare, Vita, Carswell, Thompson, Lukic, Yousry, Rudge, Mead, Collinge, Thornton, 2011). Besides, other DTI measures computed from DWI, such as fractional anisotropy (FA), mean diffusivity (MD) and radial diffusivity (RD) were used to assess the relevance of the putamen as a biomarker in the diagnosis of CJD (Hyare, Vita, Porter, Simpson, Ridgway, Mead, Rudge, Collinge, Thornton, 2013, Hyare, Vita, Ridgway, Porter, Rudge, Yousry, Mead, Collinge, Thornton, 2013).

Despite the promise of quantitative biomarkers, clinical diagnosis still relies only in the qualitative evaluation of MRI scans.

To address this limitation we proposed a framework that aims to (i) extract quantitative imaging biomarkers from MR images, (ii) show that those are fit for the diagnosis of CJD in its earlier stages, and (iii) diagnose and stratify the subjects according to severity stages of symptoms design based on a widely used clinical severity measure, the MRC Prion Disease Rating Scale Thompson et al. (2013a).

To our knowledge, this study is the first attempt to use quantitative features extracted from MR images, obtained using different MRI pulse sequences, as inputs in a classification and differential diagnosis tool, in order to capture the evolution of prion disease neuropathology over the course of the disease. Furthermore, the proposed framework also allows to assess disease severity using imaging data.

By taking advantage of tailored imaging biomarkers for classification, we believe that the proposed method can be used to achieve an early diagnosis of CJD. This it is particularly useful to improve early patient recruitment to clinical trials and/or CJD studies, and monitoring disease progression during the trial.

## 2 Materials and methods

### 2.1 Feature extraction

**Fig. 1:**
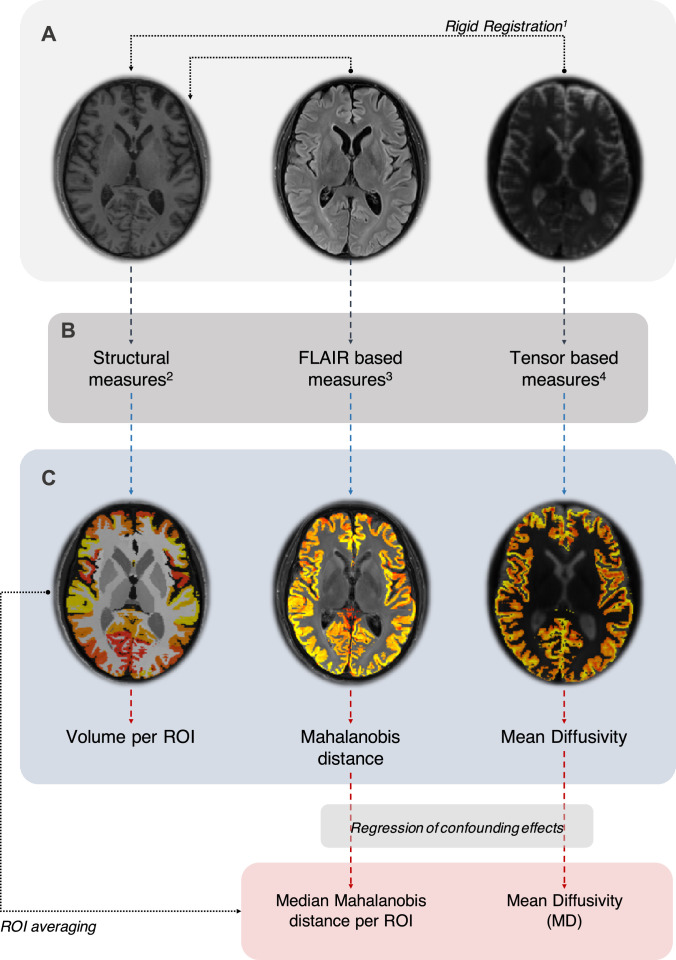
Representation of the framework adopted for feature extraction. A: data preprocessing step, including rigid registration using (1) *NiftyReg* Modat et al. (2014). B: Feature extraction per MRI sequence, applying (2) GIF algorithm (Cardoso et al., 2011) to T1, using (3) BaMoS algorithm to extract the intensity distributions of FLAIR (Sudre et al., 2015), and computing the diffusion tensor from DWI. C: Quantitative features computed from the images obtained in the section B of the framework.

For each subject, we extracted quantitative features from the three MRI pulse-sequences available in the dataset, which provide complementary information about brain microstructural changes caused by CJD. The framework, Fig. 1, consists of three sections: (A) data pre-processing, including artefact correction steps such as bias field correction, reduction of the partial volume effect, and correction of the effects of eddy currents in DWI scans; (B) and (C) specific feature extraction and quantification according to each MRI sequence. In section (A) both DWI and FLAIR scans were rigidly registered to T1w scans using the *NiftyReg* open-source software (Modat et al., 2014). We regressed out the impact of confounding effects, such as age and the total intracranial volume, by comparison with a healthy population. This correction is applied *a priori* to all the features extracted from different sequences.

To identify nerve cell loss and consequently atrophy of cortical and deep GM areas, we extracted volumetric information from T1w MRI scans using automated region of interest delineation. The Geodesical Information Flows (GIF) (Cardoso et al., 2015) algorithm, that relies on multi-atlas segmentation propagation, was used to parcellate the brain into multiple regions. The volume of each 128 individual brain region is then computed.

Hyperintensity abnormalities visible on FLAIR images need to be carefully considered since the degree and distribution of these histological changes tend to vary significantly among the different time of scanning (Chung, Williams, Ritchie, Williams, Changani, Hope, Bell, 1999, Zeidler, Sellar, Collier, Knight, Stewart, Macleod, Ironside, Cousens, Colchester, Hadley, Will, 2000). To characterise the degree abnormality in each subject’s brain, we considered as a feature the distribution of signal intensities in GM tissues in FLAIR images. Using the Bayesian Model Selection (BaMoS) algorithm (Sudre et al., 2015), we automatically segmented the normal and abnormal appearing tissue types. Knowing that CJD mainly causes lesions in the GM tissues, we computed the Mahalanobis distance (Geoffrey McLachlan, 2004), between the normal appearing WM intensity distribution and the GM intensities for each region of interest as defined by T1w derived parcellation. The Mahalanobis distance per region, d_M(GM,WM)_ is computed as

(1) $$\sqrt{{\left(\mu_{\text{GM}}-\mu_{\text{WM}}\right)}^{T}\cdot S_{\left(\text{GM},\text{WM}\right)}^{-1}\cdot \left(\mu_{\text{GM}}-\mu_{\text{WM}}\right)}\text{,}$$

where *μ*_GM_ is the mean of intensities in each GM region, *μ*_WM_ is the mean intensity of WM tissue after excluding the lesions detected as outliers, and S_(GM,WM)_ corresponds to the covariance between the two intensities distributions.

The obtained values are a quantitative measure of signal abnormalities in GM and they can be used as a feature with the assumption that the larger the amount of hyper-intensity in a given region of interest, the larger the Mahalanobis distance. The assessment of the hyper-intensities in the brain mimics the clinical practice, in which CJD is typically diagnosed based on the presence of these signal abnormalities.

The most typical brain microstructural change caused by CJD is vacuolation, or spongiosis. Spongiosis can result from abnormal membrane permeability and increased water content within neuronal processes; however, the molecular mechanisms behind vacuolation are still unclear (Soto and Satani, 2011). Spongiosis is visible in DWI scans as an increase in the diffusion signal and it can be quantified using the MD measurements. We initially processed the DWI scans using the *NiftyFit* pipeline, described in Melbourne et al. (2016), in which MD measurements are computed according to (Le Bihan et al., 2001). The median MD value per ROI is computed and used as feature.

### 2.2 Feature selection

In the literature, common approaches for imaging classification when applied to neurodegenerative diseases usually rely on a consistent set of features among subjects. Most neurodegenerative diseases follow a common spatial pattern among subjects and across time. However, these methods are not suitable for the characterisation of CJD, as the high heterogeneity of the disease yields no consistent spatial pattern of features in the brain, neither a defined pattern of events related to disease progression.

We then hypothesized that the disease does not follow a spatial pattern in the brain. Alternatively, we assumed the imaging biomarkers can become abnormal in any location in the brain, without following a particular order. The quantification of abnormality rather than its location is thus used to infer the progression of the disease. To characterise the amount of abnormality of signal for the different types of feature, we implemented a framework previously validated with IPD data (Canas et al., 2018b), in which the different features were converted into z-scores by comparison with measurements obtained from a population of healthy subjects. The absolute z-scored values are then ranked per feature type and only the highly ranked features are considered for subsequent learning and inference stages. In this study, we selected the 15 higher ranked regions of interest per subject for each sequence independently. As a result, the array of features $X={\left[x_{1,1},\cdots ,x_{N,F}\right]}^{N\times F\times m},$ where *N* is the number of subjects, *F* refers to the 15 selected features and *m* is the number of MRI sequences considered. We decided to use 15 brain regions since it does correspond to 12.5% of the subjects sample and consequently the optimisation problem becomes well-posed. As a consequence, only regions of the brain that most differ from the healthy control sample are kept for each subject, and the resulting sets of feature are subject-specific.

### 2.3 Model

In order to characterise the disease status of each subject using the available multi-source features, we designed a Bayesian framework to find the function that best fits the relationship between imaging features and the subjects’ diagnosis. Bayesian frameworks, such as Gaussian Process (GP), are particularly useful to study CJD, since they allow robust modelling even in highly uncertain or incomplete datasets. GP is also commonly used to perform long term predictions, and has been shown to improve performances as the number of samples increases (Roberts et al., 2012). Since GP is a probabilistic model, it provides an estimation of the probability of a subject being assigned to a certain class. Note that the class probability estimations can be used as a measure of the confidence of the predictions, which can be useful in a clinical context as a proxy of the diagnosis confidence and as an indicator of subjects’ prognosis.

We implemented a non-parametric kernel-based model M, as follows:

(2) $$\begin{array}{c} M:y=f(X)+\varepsilon ,\\ f∼\mathrm{GP}\left(\mu_{f};K+I\sigma_{n}^{2}\right),\;\varepsilon ∼N\left(\mu_{\varepsilon };\sigma_{\varepsilon }\right) \end{array}$$

This model was used to predict the probability of the outcome *y*  ∈  Y, for a subject i={1,…,N}, given a set of biomarkers **X**  ∈  X feature space. For the binary discriminative case, such as subjects’ diagnosis, the output of the regression model M is transformed into a class probability using a cumulative density function, also called as *probit* likelihood function, which converts its argument, which can lie in the domain (−∞,∞), into the range [0, 1], guaranteeing a valid probabilistic interpretation. Therefore, the posterior probability of each class C for a subject *i* is then given by Eq. (3), where ***Φ(.)*** denotes the cumulative density function of the standard normal distribution (Rasmussen and Williams, 2004).

(3) $$p\left(y_{i}|f\left(x_{i}\right)\right)=\Phi \left(y_{i}f\left(x_{i}\right)\right)=\int_{-\infty }^{y_{i}f\left(x_{i}\right)}N\left(x\;|0,1\right)\;dx$$

For the purposes of subjects diagnosis, the likelihood of $p(y_{i}|f\left(x_{i}\right))$ is a cumulative density function, therefore the posterior (Eq. (4)) is analytically intractable. To address this issue, we used the expectation propagation algorithm (EP) Minka (2001) to approximate the likelihood by a local likelihood approximation (Eq. (5)).

(4) $$p\left(f|X,y\right)=\frac{1}{Z}p\left(f|X\right)\prod_{i=1}^{N}p\left(y_{i}|f_{i}\right)$$

(5) $$p\left(y_{i}|f_{i}\right)≃t_{i}\left(f_{i}|{\tilde{Z}}_{i},{\tilde{\mu }}_{i},{\tilde{\sigma }}_{i}^{2}\right)\equiv {\tilde{Z}}_{i}N\left(f_{i}|{\tilde{\mu }}_{i},{\tilde{\sigma }}_{i}^{2}\right)$$

where ${\tilde{Z}}_{i},{\tilde{\mu }}_{i},{\tilde{\sigma }}_{i}^{2}$ are the site parameters, as defined in Rasmussen and Williams (2004). Therefore, the posterior p(f|X,y) can be approximated by:

(6) $$q\left(f|X,y\right)\equiv \frac{1}{Z_{\text{EP}}}p\left(f|X\right)\prod_{i=1}^{N}t_{i}\left(f_{i}|{\tilde{Z}}_{i},{\tilde{\mu }}_{i},{\tilde{\sigma }}_{i}^{2}\right)$$

The tilde notation corresponds to the local approximation of the likelihood via EP approximation, as defined in  Rasmussen and Williams (2004).

As aforementioned, the CJD phenotype is better explained by the interaction between several types of features; thus, a basis kernel function is insufficient to describe the variance of the features. Besides, it is reasonable to assume that the features extracted from one MRI sequence does not show a consistent relationship with the features extracted from other sequences, during all stages of the disease Chung et al. (1999).

**Fig. 2:**
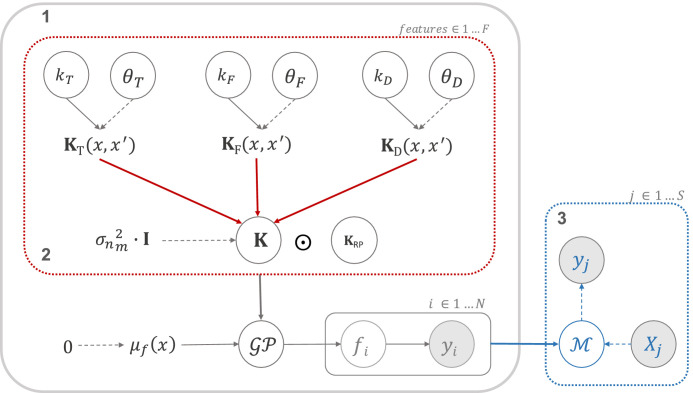
Scheme of the generative model - Eq. (2). The inner section (red line) illustrates the addition of kernel matrices computed for the features set independently. The grey section corresponds to the estimation of the hyper-parameters of the model for according to Eq. (8). The blue section corresponds to the inference stage in which a predictive label for a new subject *j* is computed using model M. (For interpretation of the references to colour in this figure legend, the reader is referred to the web version of this article.)

The inter MRI sequence relationship can thus be modelled as a multi-task paradigm – a contribution of independent functions that explain the biomarkers’ progressions. A sensible way to model a GP as a multi-task model is using an Additive GP. By implementing an Additive GP we are able (1) to express superposition of different processes contributing to the some output and (2) to improve model interpretability, since it provides information about relative weighting of different functions and their orders of interaction (Duvenaud, Lloyd, Grosse, Tenenbaum, Ghahramani, 2013, Duvenaud, Nickisch, Rasmussen, 2011). The latent function *f* in model M, Eq. (2), takes the form of $f=\sum_{m=1}^{M}f_{m},$ with $f_{m}∼\mathrm{GP}\left(\mu_{f_{m}};K_{m}+I\sigma_{n_{m}}^{2}\right),$ where *M* refers to the number of MRI sequences taken into consideration in the model (Rasmussen and Williams, 2004). The imaging biomarkers were encoded in individuals kernel matrices $K_{m},\;m\in \left\{\text{S,}\;\text{F,}\;\text{T}\right\}$ for T1w, FLAIR and MD respectively, Fig. 2. Given the kernel properties, the addition of GP with $\mu_{f}=0$ is equivalent to $f∼\mathrm{GP}(0;\sum_{m=1}^{M}K_{m}+I\sigma_{n_{m}}^{2}),$ where $\sigma_{n_{m}}^{2}$ is the noise variance per MRI sequence (Duvenaud, Lloyd, Grosse, Tenenbaum, Ghahramani, 2013, Duvenaud, Nickisch, Rasmussen, 2011). Therefore, the matrix K, Fig. 2, which encodes the imaging biomarkers, is obtained by the addition of the kernel matrices computed individually using the information extracted from the MR sequences. Note that we estimate the kernel matrices using the ranked magnitude of the feature abnormality, encoded by their *z-score*, rather than their spatial information. The consistency across features is thus with respect to their subject-specific rank of abnormality rather their spatial location. Using this approach, we construct one kernel matrix per modality before combining them using an additive GP.

Our model also accounts for the individualised pattern of each genetic mutation of the genetic form of prion disease, defined as a categorical variable in the kernel matrix $K_{RP}$. To reduce the bias introduced by the high number of genetic mutations, we grouped the subjects in two clusters according to the expected rate of disease progression associated with each mutation: (1) slow and (2) fast, defined based in the clinical knowledge^1^ about the different mutations, Eq. (7), where *r* is the rate of progression of a given subject, such as $r,r^{\prime }\in X,$ and it is defined in the features array.

(7) $$k_{\text{RP}}\left(r,r^{\prime }\right)=\left\{ \begin{array}{c} 1\;\text{if}\;r-r^{\prime }=0\\ 0\;\text{otherwise} \end{array}\right.$$

We understand that this is not actually genetic information, but rather a cluster of mutations with similar physiological behaviour. Using this kernel with the aforementioned information, we show the flexibility of our model to deal with both categorical and continuous data, such us genetic and quantitative imaging data respectively. Future work should make use of this kernel matrix to encode any other relevant genetic data, such as SNP information.

The K is lastly combined with the categorical covariance function by means of the Hadamard product, $\;\;\text{K}{\;}_{RP}⊙K$ to produce a hierarchical model, where **K** is the sum of the kernels used to encode imaging data.

The modified latent function f(X|Θ) which encodes the mutation information is $f∼\mathrm{GP}(0;\sum_{m=1}^{M}\left(K_{m}+I\sigma_{n_{m}}^{2}\right)⊙K_{RP}),$ where **Θ** is the vector of parameters of the model, which includes the hyperparameters of the kernel functions and the sample variance: $\Theta =\left[\theta_{T},\theta_{F},\theta_{D},\Sigma_{n}\right],$ where $\Sigma_{n}=\sum_{m=1}^{M}\sigma_{n_{m}}^{2}I,\;m\in \left\{\text{T,}\;\text{F,}\;\text{D}\right\}$ is the noise variance per MRI sequence.

The estimation of y_*_ requires to find the best hyperparameters of each kernel covariance function. The hyperparameters **Θ** of the kernel functions are estimated via the maximisation of the marginal likelihood of the model, p(Θ|M), as described in Eq. (8); i.e., the marginalisation over the kernel parameters is performed by maximum *a posteriori* algorithm (MAP), and the hyperparameters **Θ** are estimated by bootstrapping (Rasmussen and Williams, 2004).

(8) $$\begin{array}{c} \left\{\hat{\Theta }\right\}={\text{argmax}}_{\Theta }p\left(\Theta |M\right)=\\ {\text{argmin}}_{\Theta }\left[-\log p\left(M|\Theta \right)+\log p\left(\Theta \right)\right] \end{array}$$

The full model is illustrated in Fig. 2.

### 2.4 Subjects diagnosis

In order to diagnose symptomatic subjects among healthy controls, we modified the model M to estimate the best predictions under the form of class probabilities for $y=\left\{C_{1},\cdots ,C_{C}\right\},C\in \left\{0,1\right\},$ where 0 denotes the healthy controls and 1 denotes the symptomatic subjects. For the binary discriminative case, such as subjects’ diagnosis, the output of the regression model M is transformed into a class probability using a *probit* likelihood function. Therefore, the posterior probability of each class C for a subject *i* is thus given by Eq. (3), where **Φ**(.) denotes the cumulative density function of the standard normal distribution (Rasmussen and Williams, 2004).

The imaging biomarkers are encoded in $K_{m}$ through the use of a squared exponential kernel function (SE) with hyperparameters $\theta =\left[\sigma_{f}^{2},l^{2}\right],$ where $\sigma_{f}^{2}$ is the signal variance and *l*^2^ is the length scale (Rasmussen and Williams, 2004). The SE function is widely-used within binary classification problems, especially for its main assumptions: smoothness and stationarity.

(9) $$k_{\text{SE}}\left(x,x^{\prime }|\theta \right)=\sigma_{f}^{2}\exp\left(-\frac{1}{2}\frac{{\left(x-x^{\prime }\right)}^{2}}{l^{2}}\right)$$

In the final section of the proposed model, illustrated in Fig. 2 by the blue section, we used the optimised model M to estimate the predictive label y_*_ for a new subject^2^ j={1,⋯,S} from the testing and validation sets. The predictive probability is given by Eq. 10. Similarly, the mean $E_{q}$ and variance $V_{q}$ of function f_*_ is computed using Eqs. (11) and (12) respectively.

(10) $$\begin{array}{c} q(y_{*}=1|\text{X},y,x_{*})=\\ \Phi \left(\frac{k_{*}^{T}{\left(K+\tilde{\Sigma }\right)}^{-1}\tilde{\mu }}{\sqrt{1+k\left(x_{*},x_{*}\right)-k_{*}^{T}{\left(K+\tilde{\Sigma }\right)}^{-1}k_{*}}}\right) \end{array}$$

where $\tilde{\Sigma }$ is a diagonal matrix with $\Sigma_{n}=\left\{\sigma_{n_{m}}^{2},\;m\in \left\{\text{T,}\;\text{F,}\;\text{D}\right\}\right\}$. Note also that the $\tilde{\mu }$ is the site parameter defined in Eq. (5). The tilde notation refers to the local approximation used in the EP algorithm as defined in Rasmussen and Williams (2004).

(11) $$E_{q}\left[f_{*}|X,y,x_{*}\right]=k_{*}^{T}{\left(K+\tilde{\Sigma }\right)}^{-1}\tilde{\mu }$$

(12) $$V_{q}\left[f_{*}|X,y,x_{*}\right]=k\left(x_{*},x_{*}\right)-k_{*}^{T}{\left(K+\tilde{\Sigma }\right)}^{-1}k_{*}$$

The analysis of the latent models that compose f_*_ provide the information about the best combination of features to diagnose prion disease.

### 2.5 Subjects stratification

Being able to diagnose CJD at the early stages of the disease could increase participation in clinical trials, which is currently challenging as patients usually die in less than 12 months from diagnosis (Bradford et al., 2014). Therefore, the prediction of the time to clinical onset of IPD patients is one of the aims of this study.

The proposed model does not give a continuous measure of the time to onset, in years. Nevertheless, the stratification of the subjects according to the severity of symptoms, or the proximity to clinical onset stage, can be interpreted as the subject’s prognosis. For that purpose we adapted the generative model 2 to predict the stage of the disease for a subject *i* given the set of features **X**. The estimated probabilistic class provides a clinical input regarding the severity of symptoms of prion disease. We implemented a multi-class classification GP based on individualised likelihood factors computed for the target classes defined by $y_{i}=\left\{C_{1},\ldots ,C_{C}\right\},C\in \left\{1,\ldots ,5\right\}$ for the subject *i*. These classes correspond to the five predefined stages of the disease: (1) healthy control (HC), (2) asymptomatic subjects (Asym.), (3) subjects with an MRC Scale score of 20 i.e., asymptomatic^3^ or early symptomatic but no accrued neurodisability, within one year inside of the clinical onset window (CO), (4) to (5) symptomatic subjects divided in 2 severity quantiles according to their MRC Scale scores (Thompson et al., 2013b). The MRC Scale is a functional composite scale measured as a 0–20 score developed using item-response modelling (Thompson et al., 2013a). Items rated include memory and orientation; continence; self care; mobility; and communication.

Due to the number of classes under consideration, *f_i_* is a vector $f_{i}={\left[f_{i}^{1},\ldots ,f_{i}^{5}\right]}^{T}$. The multi-class classification is performed by means of a multinomial probit likelihood, as defined by Eq. (13), where the auxiliary variable *u_i_* is distributed as $p\left(u_{i}\right)=N\left(u_{i}|0,1\right)$.

(13) $$p\left(y_{i}|f_{i}\right)=E_{p(u_{i})}\left\{\prod_{j=1,j\neq y_{i}}^{5}\Phi \left(u_{i}+f_{i}^{y_{i}}-f_{i}^{j}\right)\right\}$$

Analogous to binary classification, the posterior of a multiclass GP is analytical intractable, thus it requires the approximation of the likelihood. To keep the consistency across classification tasks, we used EP algorithm. However, in case of binary GP the estimation of the tilted distribution (defined in Eq. (5)) requires solving one-dimensional integrals, and assuming the *probit* likelihood function, these univariate integrals can be computed efficiently without numerical quadratures (Minka, 2001, Rasmussen, Williams, 2004). For the multiclass paradigm the solution is more complex, since we need to evaluate the multi-dimensional integrals (Riihimäki et al., 2013). The approximation of the tilde variables can be done by Laplace approximation (LA) (Rasmussen and Williams, 2004). The problem with the LA approach is that the mean is replaced with the mode of the distribution and the covariance with the inverse Hessian of the log density at the mode. Because of the skewness of the tilted distribution caused by the likelihood function, the LA method can lead to inaccurate mean and covariance estimates in which case the resulting posterior approximation does not correspond to the full EP solution.

The marginal likelihood is then approximated via a nested expectation propagation (nEP), which does not require numerical quadratures or sampling to estimate the predictive probabilities, as detailed in Riihimäki et al. (2013).

In this particular case of the model M, the imaging biomarkers are encoded using a linear combination of linear logistic kernel functions (Eq. (14)), *b* is the intercept of the linear part and *a* is the regression coefficient of the linear part. This function is used to encode the variance of the biomarkers over the different stages of the disease. In previous studies focusing on neurodegenerative diseases, it has been demonstrated that imaging biomarkers show a logistic evolution over time (Canas et al., 2018a).

(14) $$\begin{array}{c} k_{\text{LINLOG}}\left(x,x^{\prime }|\Theta \right)=H\left(x\right)\Sigma_{f}H{\left(x^{\prime }\right)}^{\text{T}}\\ \Sigma_{f}=\text{diag}\left[\sigma_{f_{1}}^{2},\cdots ,\sigma_{f_{N}}^{2}\right]\\ H\left(x\right)={\left[h\left(x_{1}\right),\ldots ,h\left(x_{N}\right)\right]}^{\text{T}}\\ h\left(x\right)=({\text{logit}}^{-1}\left(ax+b\right)-0.5) \end{array}$$

Similarly, to the model described in Section 2.4, the function *f* is defined as $f∼\mathrm{GP}(0;\sum_{m=1}^{M}K_{m}⊙K_{RP}+I\sigma_{n_{m}}^{2}),$ where **Θ** is the vector of parameters of the model, which includes the hyperparameters of the kernel functions and the sample variance: $\Theta =\left[\theta_{m},\Sigma_{n}\right],$ where $\Sigma_{n}=\sum_{m=1}^{M}\sigma_{n_{m}}^{2}I,\;m\in \left\{\text{T,}\;\text{F,}\;\text{D}\right\}$ is the noise variance per MRI sequence, and $\theta_{m}=\left[a_{m},b_{m},\Sigma_{f}^{2}\right]$.

The predictive probability for a new subject *j*, given the optimised model, is given by an extension of Eq. (11), detailed in Riihimäki et al. (2013).

### 2.6 Differential diagnosis

Lastly, due to its rarity, CJD is commonly mistaken for other types of dementia, which results in a higher rate of undiagnosed subjects, and patients who present at a more advanced disease stage. Improving early diagnosis could permit (1) a more effective management of the disease symptoms and (2) planning for end of life and (3) recruitment to clinical trials. We adapted the model M to be used as a differential diagnosis tool, and applied it to identify CJD among another form of dementia: young onset Alzheimer’s disease (YOAD).

As described in Section 2.5, we computed individualised likelihood factors for the target classes defined by $y_{i}=\left\{C_{1},\ldots ,C_{C}\right\},C\in \left\{1,\ldots ,4\right\}$ for the subject *i*, where (1) corresponds to healthy controls, (2) to IPD, (3) sCJD and (4) YOAD. Analogous to subject stratification task, Eq. (14) was used to encode the imaging biomarkers.

### 2.7 Model evaluation

We evaluated the performance of model M in terms of the robustness and accuracy of the predictions. The robustness of the estimations and the stability of the results were assessed through bootstrapping.

The effectiveness of the subjects diagnosis was assessed using sensitivity, specificity, accuracy and false rate of discovery (FDR) (Sokolova and Lapalme, 2009). The receiver operating curves (ROC) and the area under the curve (AUC) were computed using the formulation for ROC graphs proposed in Fawcett (2006).

Both subjects stratification and differential diagnosis analyses were performed as a multi-task classification using unbalanced classes. Due to the multi-task paradigm and the unbalanced nature of the data, we use macro-averaging measures, generalised from the measures for binary classification evaluation (Sokolova and Lapalme, 2009). The averaging accuracy *Acc* (Eq. (15)), macro-recall *R_M_* (Eq. (16)), macro-precision *P_M_* (Eq. (17)) are computed for model evaluation. Note that the *tp_c_* is the true positive for the class $C_{c},$ and *fp_c_* false positive, *fn_c_* false negative, and *tn_c_* true negative counts respectively. The predicted label for each subject was obtained based on the class with highest probability among all the possible classes.

(15) $$Acc=\frac{\sum_{c=1}^{l}\left(\frac{tp_{c}+tn_{c}}{tp_{c}+tn_{c}+fp_{c}+fn_{c}}\right)}{l}$$

(16) $$R_{M}=\frac{\sum_{c=1}^{l}\left(\frac{tp_{c}}{tp_{c}+fn_{c}}\right)}{l}$$

(17) $$P_{M}=\frac{\sum_{c=1}^{l}\left(\frac{tp_{c}}{tp_{c}+fp_{c}}\right)}{l}$$

To evaluate the probabilistic predictions performance of the model, we also computed the **multiclass logarithmic loss** (Eq. (18)):

(18) $$\log\left(L\right)=-\sum_{c=1}^{C}\log\left(p_{o,c}\right)$$

where *p*_*o,c*_ is the probability of observation of class C.

### 2.8 Data and experiments

#### 2.8.1 Data processing

The data used in this study were obtained from the National Prion Monitoring Cohort (NPMC). NPMC (2008-) is a prospective observational interval-cohort study of patients with any form of prion disease in the UK or willing to travel to the UK. It includes regular follow-up clinical and psychological assessments of sCJD patients and patient with IPD and their relatives who may be known carriers of *PRNP* gene mutations, at-risk but not had a genetic test, or healthy controls. The current dataset comprises (a) symptomatic patients with confirmed prion disease diagnosis, for both the inherited and sporadic forms of the disease, defined in the following sections as IPD and sCJD respectively; (b) healthy subjects without a clinical diagnosis of IPD who carry *PRNP* gene mutations and are therefore at increased risk of disease in the future, defined in this study as asymptomatic subjects; (c) healthy individuals without either prion disease or increased risk, defined as healthy controls (HC). From the sample aforementioned detailed, we defined a group composed by the subjects at clinical onset (CO) (subjects within one year of a clinical diagnosis and an MRC scale of 20 or less), in order to examine specific brain changes occurring during the clinical onset. To avoid the overlap of criterias used to defined both the CO and IPD groups, the IPD group is composed only by symptomatic subjects with an MRC scale equal or lower than 20, in which the scans were acquired more than one year after clinical onset. Data from all 125 subjects include MRI scans, neurological and neuropsycological assessment and scoring using the MRC Scale Thompson et al. (2013a).

MRI was acquired using a Siemens Magneton Trio (Siemens, Erlanger, Germany) 3 Telsa with the conventional body coil for transmission and a 32-channel head-only receive coil. Structural imaging used 3D T1-weighted images (T1w) MPRAGE sequence with repetition time 2.2 s, echo time 2.9 ms, inversion time 900 ms, echo spacing 6.7 ms, flip angle 10^∘^, matrix size 256 × 256 × 208, voxel size 1.1 × 1.1 × 1.1 mm. 2D Axial FLAIR were acquired using a standard clinical FLAIR-TSE sequence with a voxel size of 0.9 × 0.9 × 5.2 mm. The diffusion weighted imaging (TR/TE 9500/93ms) were acquired using 64 non-colinear directions at $b=1000\text{s}/{\text{mm}}^{2}$ and 8 images with b = 0. For all subjects a T1w image was acquired as well as either a FLAIR, a DWI, or both.

The YOAD subjects used for the differential diagnosis experiment are part of a larger study of young onset Alzheimer’s disease, for which ethical approval was obtained from the National Hospital for Neurology and Neurosurgery Research Ethics Committee. T1w and DWI images were acquired using the same scanner. The T1w pulse sequence was identical to the one used for the other data sets. The DWI acquisition however differ for the two datasets. Multiple shells were acquired for the YOAD dataset. We here used the shell that had the most similar b-value (b = 700) than the one used for the prion data acquisition (b = 1000). We acknowledge this limitation as a potential bias. For YOAD subjects no FLAIR images were acquired.

The sample’s demographics are detailed in Table 1.

**Table 1:** Demographic and imaging information of subjects in the baseline of NPMC and YOAD database, included in this study. The full model is the model trained using only the subjects with all the three MRI sequences available. The number of mutations details the number of different mutations existing among the subjects.

	Groups		Age (years)	Full Model (Male)	T1w (Male)	FLAIR (Male)	DWI (Male)	#Mutations
	Healthy Controls		48.2 (23.3 - 75.2)	29 (16)	31 (16)	29 (16)	26 (16)	—
**Diagnosis**	IPD		47.7 (24.9 - 61.4)	16 (11)	30 (18)	21 (12)	18 (11)	8
	Sporadic CJD		63.7 (53.3 - 76.7)	17 (10)	28 (15)	20 (11)	17 (10)	—
	YOAD		61.0 (48.0 -74.0)	—	32 (10)	—	32 (10)	—
**Stratification**	Asympt. IPD		42.7 (19.5 - 72.3)	22 (6)	31 (11)	29 (16)	22 (6)	6
	Clinical Onset		50.7 (41.6 - 65.2)	4 (3)	5 (3)	5 (3)	4 (3)	3
	*Stage I*	IPD	44.5 (24.9 - 61.3)	15 (9)	20 (11)	20 (12)	16 (10)	7
		sCJD	63.9 (54.3 - 75.7)	5 (3)	5 (3)	7 (4)	5 (3)	—
	*Stage II*	IPD	26.1	1 (0)	3 (2)	1 (0)	1 (0)	1
		sCJD	62.5 (53.3 - 71.5)	11 (6)	21 (12)	19 (11)	19 (10)	—

#### 2.8.2 Experiments

In order to assess the performance of the proposed framework, we designed four experiments to evaluate: (1) The feature selection strategy, (2) the subjects diagnosis classification performance, (3) the subjects stratification sensitivity and (4) the differential diagnosis capabilities. In experiments (2) to (4), we trained the model using 75% of the overall sample while keeping the input ratio between the different groups. The testing set corresponds to the remaining 25% of each sample. The hyperparameters of the model were optimised using an open-source toolbox 4. In order to obtain a robust evaluation, we applied a cross-validation scheme with 500 runs for all experiments. Lastly, to understand what is the best set of MRI sequences for each task, we analysed the predicted labels obtained with the latent functions of our model. The latent functions consist of functions where individual likelihood factors depend on multiple latent variables. In this study, the latent variables are represented by the different kernel matrices that encode the imaging features. By comparing the predictive accuracy obtained by the latent functions, we can directly compare the performance of the model with different combinations of features and, consequently, infer what is the best set of features for a specific task. This analysis was performed for the experiments (2) to (4).

*(1) Feature selection* As previously detailed in Section 2.8.1, we grouped the subjects based on their prion disease subtypes and the existence of clinical symptoms. Using a non-parametric statistical test, we examined whether the selected features could differentiate the groups. We also implemented a multi-comparison test to investigate which set of features allows the best differentiation of the groups. The resulting p-values were corrected for multiple comparison using the Bonferroni method. All the subjects were included in this experiment, independently of the number of the MRI sequences available, since the features extraction and selection were performed separately for each set of features.

*(2) Subjects diagnosis* We evaluated the ability of our model to correctly diagnose subjects with the two subtypes of CJD independently. We performed the diagnosis of the subtypes of CJD separately in order to avoid the confounding effects related to the specific features of each subtype. For both IPD and sCJD subtypes, we classified only the subjects clinically labelled as symptomatic. Only the subjects with the three MRI sequences have been included in this experiment, due to the design of model M which requires joint modelling of the three set of features. Note that for IPD subjects the rate of progression varies as mentioned; whereas the sCJD subtype is always considered as having a fast progression. Further, to avoid missing information for healthy controls, these were randomly assigned a value between 1 and 3 to encode a virtual rate of progression.

A squared exponential Support Vector Machine (SE-SVM) was also used to perform the subjects diagnosis. The results of SE-SVM were compared with the our model and considered as baseline.

*(3) Subjects stratification* Using cross-sectional data, we trained the model to perform subjects’ stratification on the HC, asymptomatic subjects, IPD and sCJD. Note that in this specific experiment the sCJD and IPD subjects are jointly classified, based on their MRC Scale score. Contrarily to experiment (2), we neglected the impact of the disease rate progression modelled by the categorical kernel $K_{\text{RP}}$. Here, we assumed that sCJD patients show a disease progression rate analogous to the IPD subjects with the fastest progression rate. The motivation to include the sCJD subjects in this experiment is the small sample size of IPD patients with more severe symptoms – MRC scale lower than 14 (stage II, Table 1).

Therefore, we considered 5 classes: (1) HC, (2) stable Asymp., (3) CO – subjects at clinical onset (early symptomatic subjects with MRC Scale score equal to 20 and within an year window after conversion), (4) S-I – Symptomatic subjects with MRC Scale score between 19 and 15, and finally (5) S-II – Symptomatic subjects with MRC Scale score below 14. Only 2 subjects had an MRC Scale score below 10, and they were included in the last group. Similarly to experiment (2), to model the different rates of progression of the subtypes of CJD and of the IPD mutations, we included the $K_{\text{RP}}$ in the model.

*(4) Differential diagnosis:* Lastly, we compared the CJD subtypes against a clinically releted form of dementia. For this experiment, we included the HC, IPD, sCJD and YOAD groups. The asymptomatic subjects have been excluded to avoid the presence of confounding effects during the training of the model. Asymptomatic subjects form indeed a heterogeneous group as individuals can be days or decades from clinical onset. The features used to characterise YOAD subjects were obtained from DWI and T1w MRI scans, which have been processed using the framework detailed in Section 2.1 and 2.2. Similarly, only DWI and T1w imaging features were considered to characterise CJD subjects. Note that the feature selection section of this framework was tailored to maximise the information related to CJD symptoms; thus, the features do not encode the spatial pattern that characterises YOAD.

## 3 Results

The proposed framework was used to extract the individual features from T1w, DWI and FLAIR, which were then used as input feature in a classification algorithm aiming either at the diagnosis or staging of the individuals. The resulting features are detailed in Section 3.1. The results of the subjects’ diagnosis for both IPD and sCJD are summarised in Section 3.2. We present in Section 3.3 the results of the subjects’ stratification experiment. The effectiveness of the differential diagnosis tool is presented in Section 3.4.

### 3.1 Feature extraction and selection

We evaluate the statistical significance of the features extracted before feature selection. Table 2 shows the brain regions and their respective p-value computed using a two sample *t*-test comparing the different groups with the healthy population. Neither the Asymp. nor the CO presented any significant difference in features compared to the healthy controls. The features extracted from FLAIR are insufficient to identify brain regions that are relevant to diagnose CJD.

**Table 2:** Evaluation of the statistical significance of the imaging biomarkers, before feature selection. The two sample *t*-test was used to identify which brain regions show significant differences between symptomatic subjects and the healthy population. The p-values indicate the test rejection of the null hypothesis at 5% significance level, considering the Bonferroni correction.

DWI		
IPD	**Brain Regions**	**P-value**
	Right cuneus	1.74E-5
	Left central operculum	2.48E-5
	Right anterior cingulate gyrus	2.91E-5
	Right inferior frontal gyrus	3.54E-5
	Right angular gyrus	3.61E-5
sCJD	Right frontal operculum	1.03E-6
	Left entorhinal area	1.05E-6
	Cerebellar Vermal Lobules VI-VII	1.99E-6
	Cerebellar Vermal Lobules I-V	2.35E-6
Structural		
IPD	**Brain Regions**	**P-value**
	Left cuneus	6.01E-8
	Right cuneus	7.55E-5
	Left central operculum	5.03E-6
sCJD	Left cuneus	2.45E-9
	Right cuneus	1.11E-6
	Left central operculum	3.89E-6
	Left hippocampus	5.00E-5
	Right hippocampus	5.49E-5

**Fig. 3:**
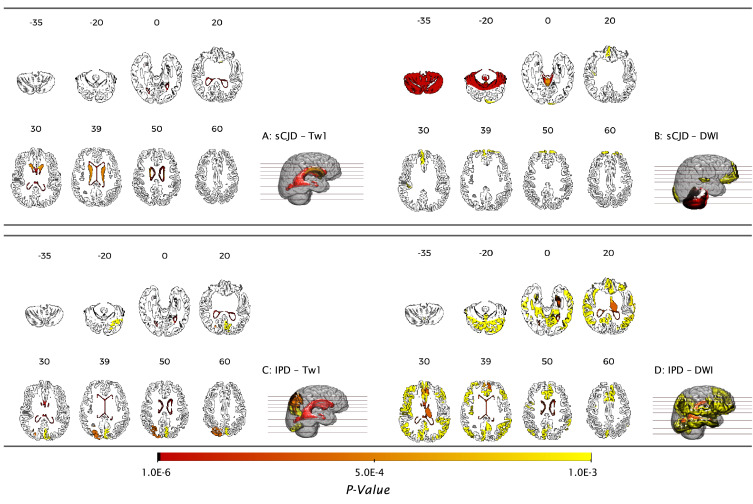
Evaluation of the statistical significance of the imaging biomarkers, before feature selection. The colour map encodes the p-value obtained from the two sample *t*-test, for each brain region showing. Seven axial slices *(zz)* (zz∈{−35;−20;0;20;30;39;50;60}) show the brain areas with significant features. A: sCJD structural features; B: DWI features extracted from sCJD data. C: T1w volumetric features extracted from IPD subjects; D: DWI features obtained from IPD scans.

Fig. 3 shows the p-values of the brain regions significantly different from the healthy population, projected into the MNI152 linear template Fonov et al. (2009) using the MRIcroGL visualisation software.^5^ The brain regions are considered as significantly different from the healthy population for the p-value $<3.79\times 10^{-04},$ presented in the Fig. 3 with light orange.

**Fig. 4:**
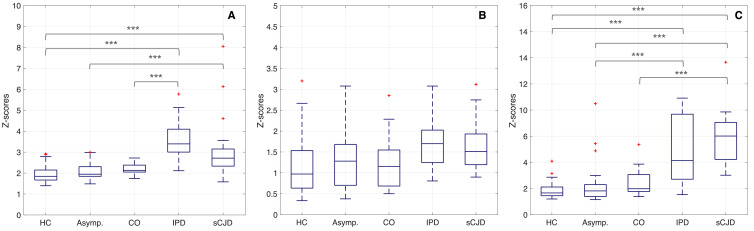
Mean of the 15 highest ranked imaging features per subject, after feature selection. A: structural features extracted from T1w scans. B: intensity based features computed using FLAIR images. C: MD computed from DWI. The red crosses represent outliers, whilst the grey asterisks represent a statistical significance of p−value<0.01. HC – healthy controls; Asym. – asymptomatic subjects; CO – clinical onset; SI – stage I and SI - stage II of the disease. (For interpretation of the references to colour in this figure legend, the reader is referred to the web version of this article.)

Due to the assumption of spatial heterogeneity of brain changes caused by prion disease, we implemented a subject-specific features extraction and selection. This approach selects the most significant features to characterise the evolution of symptoms for each subject, neglecting the spatial origin of features in the brain. Fig. 4 shows the mean of the 15 most significant features per subject and across groups.

The results of the statistical analysis performed on the features selected are detailed in Table 3. The mean of the highest ranked features extracted from both T1w and DWI are significantly different across groups, whereas the features extracted from FLAIR images have not shown statistical significance across groups.

**Table 3:** Evaluation of the statistical significance of the imaging biomarkers, after feature selection. The Kruskal–Wallis test result is shown with the null hypothesis that the sample data from each group of subjects came from the same distribution. The bold p-values indicate the test rejection of the null hypothesis at 5% significance level, considering the Bonferroni correction, $\text{p-value}<3.80^{-4}.$

	T1w	FLAIR	DWI
HC vs Asym.	0.622	0.838	0.986
HC vs Conv.	0.082	0.941	0.242
HC vs IPD	**9.92E-09**	0.004	**2.53E-06**
HC vs sCJD	**5.45E-07**	0.008	**9.96E-09**
Asym. vs CO.	0.828	0.999	0.695
Asym. vs IPD	**5.63E-08**	0.195	**7.55E-04**
Asym. vs sCJD	0.006	0.266	**8.22E-07**
CO. vs IPD	**7.32E-05**	0.166	0.065
CO. vs sCJD	0.191	0.228	**6.71E-04**
IPD vs sCJD	0.115	0.999	0.728
All groups	**2.47E-17**	6.67E-04	**2.21E-13**

HC – healthy controls; Asym. – asymptomatic subjects; CO – clinical onset; SI – stage I and SI - stage II of the disease.

Furthermore, using a multiple comparisons test we also analyzed which set of features allow differentiation of the patient groups. This experiment indicates that DWI and T1w features enable the diagnosis of sCJD vs HC with high statistical significance, whilst the T1w features identify the disease stage of IPD (vs Asym.) with highest statistical significance.

### 3.2 Subjects diagnosis

**Fig. 5:**
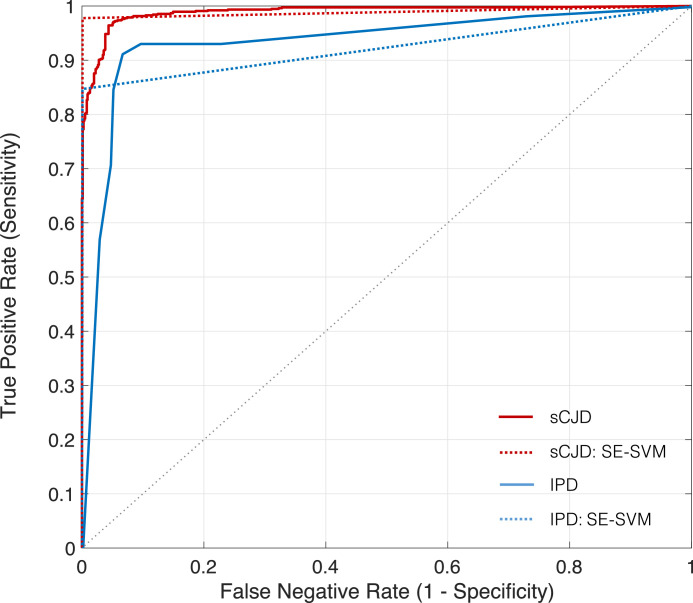
Predictive accuracy of the model for both IPD and sCJD subjects, when considering a dataset composed by the three MRI sequences. The predictive accuracy for both IPD and sCJD subjects, using squared exponential SVM (SE-SVM) is also computed using the three modalities. The ROC curves are computed considering the predicted labels of 500 iterations, as proposed by Fawcett (2006).

Fig. 5 shows the predictive accuracy of the model M (Eq. (3)) when using imaging biomarkers extracted from the three MRI sequences. The ROC curves show that the model is more effective in the diagnosis of sCJD (AUC=0.985±0.06), when compared with IPD classification (AUC=0.892±0.06) in both cases vs. controls.

To investigate the influence of each feature to the subjects’ diagnosis performances, we evaluated the accuracy of the predictive classes obtained using the latent models. Table 4 details the accuracy of the classification of sCJD patients using different sets of features. The imaging structural biomarkers extracted from FLAIR images appear to be insufficient to diagnosis sCJD subjects at the early stages of the disease, whereas the MD measures computed from DWI scans have the strongest influence in the diagnosis of sCJD, followed by the T1w imaging biomarkers.

**Table 4:** Evaluation of the full model used for subjects diagnosis, for Sporadic CJD (sCJD). The mean value and standard deviation of 500 iterations is computed for all the metrics used for performance evaluation. The AUC is computed considering the results of all iterations. The false discovery rate (FDR) is also evaluated. All the evaluation measures are presented in percentage, excepting the AUC and the L. SE-SVM stands for Support Vector Machine with squared exponential kernel.

	Accuracy	Sensitivity	Specificity	FDR	AUC	L
SE-SVM	98.82 (3.90)	97.75 (7.50)	99.90 (1.58)	0.01 (1.58)	0.99 (0.04)	1.13 ( < 0.01)
T1w	90.01 (10.34)	89.55 (16.60)	90.48 (16.76)	9.52 (0.17)	0.95 (0.10)	0.54 (0.18)
FLAIR	60.98 (15.48)	85.19 (22.29)	36.77 (29.25)	63.23 (0.29)	0.60 (0.25)	0.69 (0.01)
DWI	98.61 (4.62)	97.22 (9.23)	99.90 ( < 0.10)	0.1 ( < 0.01)	0.99 (0.01)	0.51 (0.18)
T1w + FLAIR	88.29 (13.33)	87.83 (19.33)	88.76 (20.00)	11.24 (0.03)	0.94 (0.13)	0.54 (0.19)
T1w + DWI	94.84 (8.79)	93.12 (14.47)	96.56 (10.59)	3.44 (0.05)	0.99 (0.03)	0.40 (0.22)
FLAIR + DWI	96.16 (10.13)	93.65 (16.41)	98.68 (10.22)	1.32 (0.02)	0.99 (0.06)	0.51 (0.18)
**T1 + FLAIR+DWI**	94.51 (9.96)	92.86 (15.52)	96.16 (12.21)	12.21 (0.06)	0.99 (0.06)	0.34 (0.15)

We also evaluated the predictive accuracy of the model for diagnosis of IPD subjects. Similarly to the sCJD diagnosis, the intensity based features extracted from FLAIR achieved a lower accuracy when used as single features in the model. It can be observed that including the rate of progression associated with specific mutations yields an improvement of the predictive accuracy. Note also that the full model did not necessarily achieved the best performances for all metrics, which can be justified by the introduction of noise due to the features’ interactions.

Lastly, the predictive accuracy of the SE-SVM model was also evaluated for each set of biomarkers: T1w, FLAIR and DWI. Tables 4 and  5 show that the accuracy obtained from the binary classification using SE-SVM is comparable with the predictive accuracy of our model, namely on the sCJD diagnosis. Nevertheless, our model showed a significantly lower logarithmic Loss in both tasks, which is translated in a lower uncertainty of the predictions given by our model. Therefore, even with a higher accuracy, the SE-SVM is not suitable to be used in clinical context given the uncertainty of the predicted classes.

**Table 5:** Performance of the model for IPD diagnosis. The mean value and standard deviation of 500 iterations is computed for all the metrics used for performance evaluation. We included the impact of the rate of progression (RP) of the several mutations as a categorical variable in the model. In the full model, we modelled the join contribution of the DWI, FLAIR, T1w and the RP. Accuracy, sensitivity, specificity and false rate of discovery (FDR) are shown in percentage. The comparison with Support Vector Machine with squared exponential kernel (SE-SVM) is presented.

	Accuracy	Sensitivity	Specificity	FDR	AUC	L
*SE-SVM*	91.73 (8.89)	84.68 (16.37)	99.92 (1.26)	0.08 (1.24)	0.92 (0.08)	0.78 ( < 0.01)
T1	93.70 (8.77)	93.00 (11.89)	94.40 (13.46)	5.40 (12.70)	0.95 (0.09)	0.35 (0.29)
FLAIR	56.36 (17.64)	80.73 (24.75)	31.98 (23.79)	67.82 (23.94)	0.53 (0.19)	0.70 (0.07)
DWI	77.63 (13.58)	80.40 (18.15)	74.84 (25.65)	24.93 (22.31)	0.69 (0.20)	0.69 (0.07)
T1 + FLAIR	93.48 (8.87)	93.00 (11.89)	93.95 (13.96)	5.85 (13.31)	0.95 (0.09)	0.35 (0.28)
T1 + DWI	92.53 (9.21)	93.00 (11.89)	92.05 (15.68)	7.75 (15.13)	0.94 (0.09)	0.36 (0.28)
FLAIR + DWI	70.85 (16.25)	74.22 (21.86)	67.48 (25.73)	32.32 (25.60)	0.67 (0.21)	0.69 (0.08)
T1 + FLAIR + RP	93.50 (8.87)	93.12 (11.74)	94.00 (13.93)	5.80 (13.28)	0.94 (0.09)	0.36 (0.28)
T1 + DWI + RP	93.05 (9.16)	92.85 (12.02)	93.10 (15.09)	6.70 (14.50)	0.94 (0.10)	0.37 (0.28)
FLAIR + DWI + RP	70.78 (15.74)	74.23 (20.92)	67.32 (26.16)	32.48 (26.02)	0.69 (0.21)	0.69 (0.09)
T1 + FLAIR + DWI	91.93 (9.07)	93.00 (11.89)	90.85 (15.90)	8.95 (15.37)	0.94 (0.09)	0.37 (0.28)
**T1w + FLAIR+DWI+RP**	92.45 (9.09)	93.14 (11.78)	91.90 (15.48)	7.90 (14.93)	0.94 (0.10)	0.37 (0.27)

Furthermore, the good results obtained by the SE-SVM sustained the hypothesis that subject-specific features are suitable to diagnose CJD, since these features even when used in a different classifier lead to a good identification of prion disease.

### 3.3 Subjects stratification

**Fig. 6:**
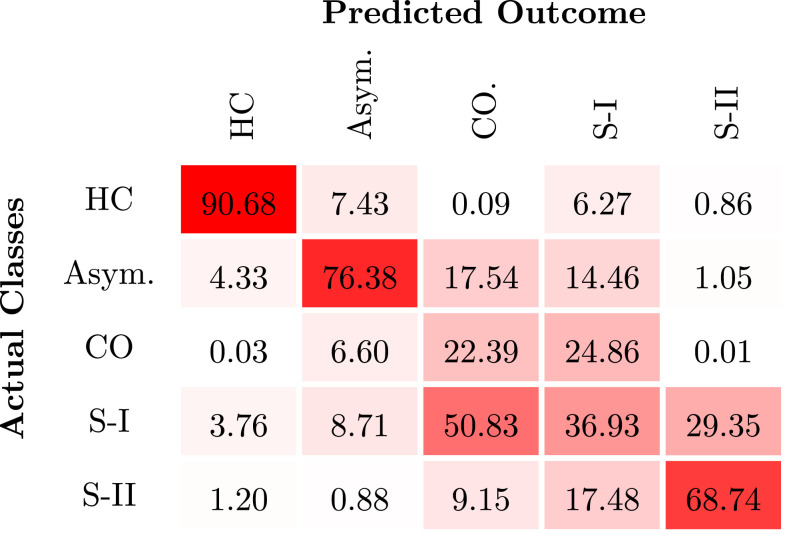
Subjects Stratification. The discrete confusion matrix was computed based on the mean of 500 iterations of the model. The values correspond to the mean percentage of subjects labelled as belonging to a given class. The intensity of the color increases with percentages. HC – healthy controls; Asym. – asymptomatic subjects; CO – clinical onset; SI – stage I and SI - stage II of the disease.

Fig. 6 shows the normalised confusion matrix for the testing set. The qualitative analysis of the confusion matrix suggests that the model is able to correctly identify the extreme stages of the disease, while being less accurate in the differentiation of the intermediate stages of the disease. The results reported in Fig. 6 are deterministic and they do not account for the fuzziness of the classes estimated, particularly for the asymptomatic stage.

**Fig. 7:**
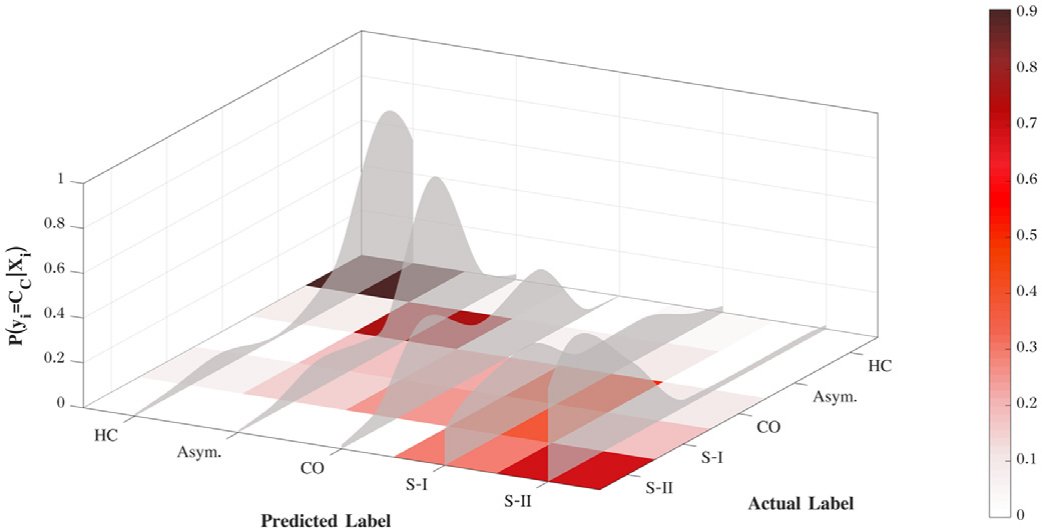
Stratification of prion disease patients using the proposed framework. The discrete confusion matrix is normalised by the number of subjects included in the classification task. The shadow area is the average distribution of probabilities per class, computed over the 500 iterations.

Fig. 7 shows the correlation between the categorical (discrete) labels and the average probability given to each class, computed through bootstrapping. The probability distribution across classes gives a more intuitive interpretation of subjects’ clinical status. For a clinical application, the probability of the prediction associated with the predicted label corresponds to the model confidence in its output.

We further investigated what is the best combination of features to achieve a good stratification of subjects, by computing the predictive classes obtained from the latent models. Table 6 presents these findings, where the average accuracy across stages is higher for the jointly modelling of the three set of features (Average Acc = 85 ± 4%) combined with the progression rate of the individual mutation. The logarithmic loss, computed across classes, is also lower for the full model (L=1.74±0.44), supporting the assumption that by using the three MRI sequences we better explain the CJD symptoms and we are able to infer the subject’s prognosis. Moreover, the low logarithmic loss translates the higher certainty of the classes correctly label, whilst the classes wrongly predicted have often higher uncertainty related to them.

**Table 6:** Performance of the proposed approach when used for disease staging. The mean value and standard deviation of 500 runs is computed for all the metrics used for multiclass evaluation. The values are presented in percentage, excepting the logarithmic Loss.

	*Average Acc*	*Precision_M_*	*Recall_M_*	L
T1w	69.43 (2.16)	4.80 (1.50)	19.96 (0.80)	3.14 (1.11)
FLAIR	69.17 (1.68)	5.24 (3.71)	20.08 (2.14)	2.60 (0.13)
DWI	69.17 (1.68)	5.24 (3.71)	20.08 (2.14)	2.60 (0.13)
T1w + FLAIR	70.26 (2.03)	6.83 (5.28)	20.67 (4.07)	2.66 (0.23)
T1w + DWI	70.26 (2.03)	6.83 (5.28)	20.67 (4.07)	2.66 (0.23)
FLAIR + DWI	69.09 (1.78)	5.09 (3.36)	19.94 (2.35)	3.84 (1.66)
T1w + FLAIR +RP	78.74 (5.11)	35.18 (13.15)	39.40 (10.45)	2.05 (0.16)
T1w + DWI + RP	78.74 (5.11)	35.18 (13.15)	39.40 (10.45)	2.05 (0.16)
FLAIR + DWI + RP	77.62 (5.60)	35.31 (17.37)	42.85 (13.26)	2.47 (0.68)
T1w + FLAIR + DWI	69.88 (1.96)	6.48 (5.81)	20.97 (4.21)	6.03 (2.01)
***T1w+FLAIR+DWI+RP***	85.39 (4.21)	57.34 (11.91)	58.83 (11.93)	1.74 (0.44)

RP – Rate of Progression.

As the model has not been re-trained for each latent model individually, the performance on the latent models that include DWI and FLAIR are very similar. This is due to their limited influence on the full model.

### 3.4 Differential diagnosis

**Fig. 8:**
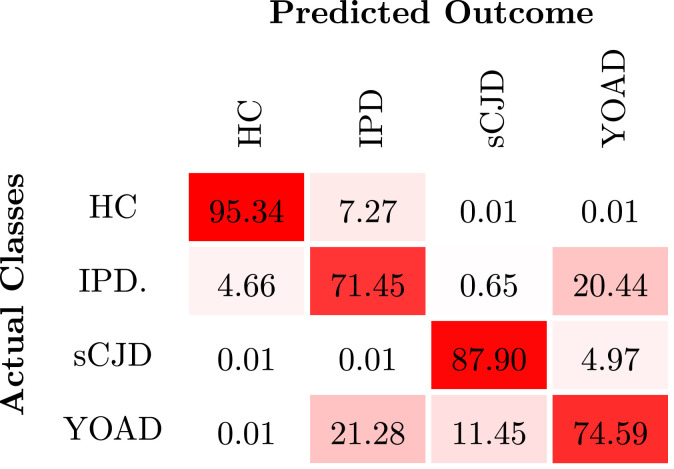
Differential diagnosis of CJD subtypes. The confusion matrix shows the mean percentage of predictive labels across the 500 runs of the model. The higher percentages of subjects classified with a given label across iterations are shown with an intense colour. HC – healthy controls; Asym. – asymptomatic subjects; CO – clinical onset; SI – stage I and SI - stage II of the disease.

By modelling the joint contribution of the three sets of features it is possible to achieve a good differentiation between the symptomatic CJD subjects and the YOAD patients, as reported in Fig. 8. The confusion matrix also shows that only approximately 8% of CJD subjects are labelled as HC. We presume that the slower rate of progression of IPD subjects and the higher number of subjects with MRC scale of 20 lead to less evident symptoms and consequently a proximity to the HC biomarkers pattern.

**Fig. 9:**
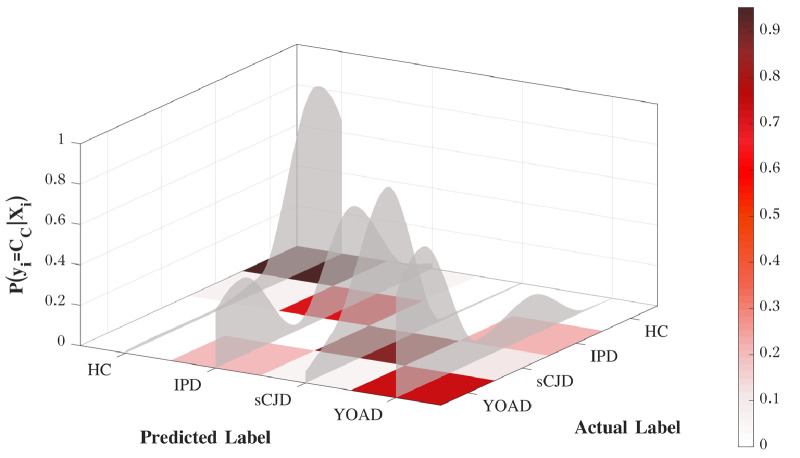
Likelihood of the predictive classes obtained from the differential diagnosis framework. The discrete confusion matrix is normalised by the number of subjects included in the classification task. The shadow area is the average distribution of probabilities per class.

The results also indicate that 75% of the YOAD subjects have been correctly labelled, showing a probability of 0.61 of being YOAD (Fig. 9); whereas the CJD subjects have shown a probability of 0.35 of being wrongly labelled as YOAD subjects, with a higher uncertainty in the differentiation of IPD and YOAD. The overlap between these two classes is explained by the lack of a specific kernel matrix to explain the spatial differences between the two diseases. Our approach only correlates the magnitude of symptoms and the correlation between the features selected to a specific form of dementia. Nonetheless, this section is an illustrative example of the flexibility of the proposed model and the possibility of being used as a differential diagnosis tool, particularly to identify CJD among other types of dementia.

**Fig. 10:**
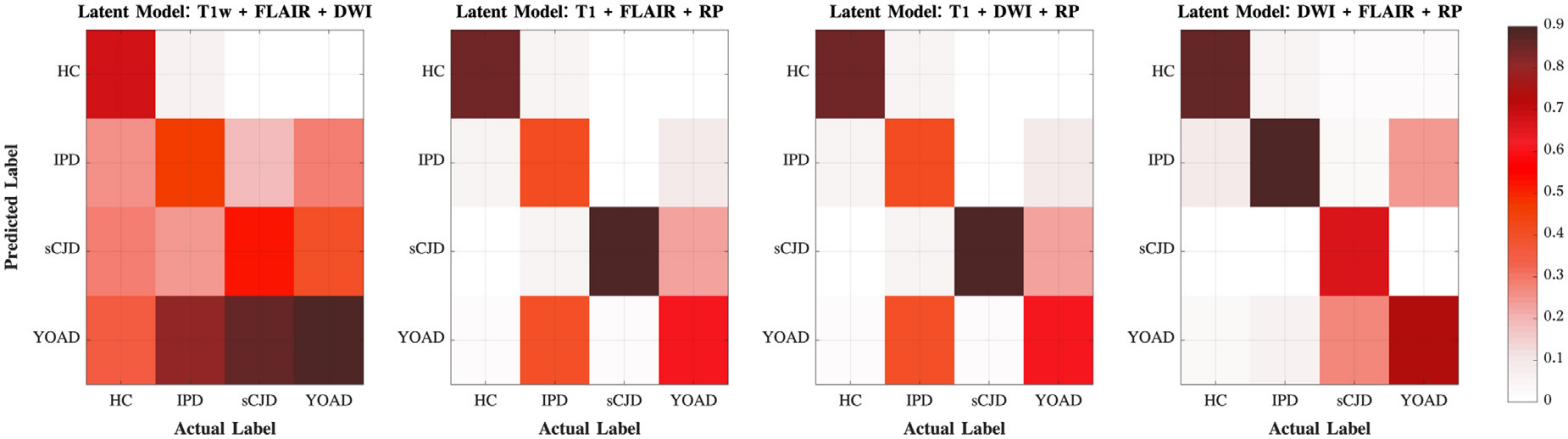
Predictive classes of the differential diagnosis task.The confusion matrices show the results of the latent models for the differential diagnosis task.

The analyses of the latent models performance show that the categorical kernel used to encode the progression of IPD mutations, highly improves the accuracy and precision of the differential diagnosis tool. Fig. 10 highlights that the combination of features from DWI and FLAIR is the most sensitive to differentiate IPD from YOAD, whereas T1w images combined either with DWI or FLAIR show better results in the identification of sCJD among YOAD patients. The micro-structural changes happening in the brain of IPD symptomatic patients visible in DWI and FLAIR can be used as the main feature to distinguish this type of CJD from other neurodegenerative syndromes.

## 4 Discussion

In this study we proposed a novel framework to extract quantitative imaging biomarkers to diagnose and stage prion disease patients. These biomarkers were also used to characterise the disease stage within a stratification task. To the best of our knowledge, this work is the first attempt to extract quantitative imaging biomarkers to be used in an automated diagnosis tool for prion related applications.

Due to the high heterogeneity of the clinical manifestations of prion diseases, it is very challenging to select useful biomarkers that may be used to comprehensively characterise all the different subtypes of prion disease and to perform an accurate diagnosis.

Previous studies have shown signs of atrophy in temporal, cingulate, frontal, parietal and occipital lobes caused by IPD (Alner et al., 2012). By using the structural biomarkers obtained via the proposed framework, we also identified statistical differences in the occipital gyrus, specifically in the cuneus, for both IPD and sCJD. The left and right hippocampus and central opercullum had been identified as meaningful regions to identify CJD, as suggested by De Vita and collaborators (Vita et al., 2015). However, we were not able to identify signs of atrophy in the temporal and parietal lobes. Our analysis also identified signal abnormalities in DWI scans. Statistical significant differences were observed in the sCJD sample, when compared with controls, in the left and right entorhinal areas, cerebellar vermal lobus I-VII. In turn, DWI signal differences were observed in the right cuneus, anterior cingulate gyrus, angular gyrus and central operculum for IPD subjects. Previous studies  (Shiga, Miyazawa, Sato, Fukushima, Shibuya, Sato, Konno, Doh-ura, Mugikura, Tamura, Higano, Takahashi, Itoyama, 2004, Young, Geschwind, Fischbein, Martindale, Henry, Liu, Lu, Wong, Liu, Miller, Dillon, 2005), have reported signal abnormalities in DWI scans in the caudate, putamen and pulvinar nuclei. Our analysis did not reveal relevant changes in those regions (Table 7).

**Table 7:** Performance of the model for the differential diagnosis. The mean value and standard deviation over 500 runs is computed for all the metrics used for performance evaluation. The average accuracy, macro precision and macro recall are shown in percentage. RP refers to the rate of progression.

	*Average Acc*	*Precision_M_*	*Recall_M_*	L
T1w	60.99 (4.97)	7.55 (6.40)	25.40 (2.90)	3.40 (1.68)
FLAIR	60.69 (4.89)	5.91 (3.66)	25.09 (1.96)	2.79 (0.26)
DWI	60.69 (4.89)	5.91 (3.66)	25.09 (1.96)	2.79 (0.26)
T1w + FLAIR	63.75 (6.08)	17.49 (13.63)	34.44 (10.81)	2.94 (0.61)
T1w + DWI	63.75 (6.08)	17.49 (13.63)	34.44 (10.81)	2.94 (0.61)
FLAIR + DWI	60.69 (4.89)	5.91 (3.66)	25.09 (1.96)	5.52 (2.46)
T1w + FLAIR +RP	80.95 (8.33)	67.06 (18.90)	63.80 (13.15)	1.51 (0.20)
T1w + DWI + RP	80.95 (8.33)	67.06 (18.90)	63.80 (13.15)	1.51 (0.20)
FLAIR + DWI + RP	85.11 (8.31)	70.39 (20.58)	71.28 (12.69)	1.52 (0.50)
T1w + FLAIR + DWI	64.26 (7.35)	15.38 (14.09)	34.24 (11.26)	5.34 (2.40)
***T1w+FLAIR+DWI+RP***	88.90 (6.89)	80.86 (11.23)	77.09 (9.28)	0.97 (0.31)

By comparing the imaging features extracted from healthy controls and symptomatic patients, we observed that CJD disease burden weighs equally on each hemisphere. Furthermore, despite the initial assumption of spatial heterogeneity of the brain changes, we identified some regions with higher prevalence among subjects with the same form of CJD (Fig. 3). These results are explained by the broad spectrum of symptoms stages found in the IPD and sCJD groups. Due to the spatial heterogeneity of the symptoms, patients at similar disease stage exhibit abnormality in non-consistent areas of their brain. Therefore, event-based models previously used to study other forms of dementia, in which a spatial homogeneity is seen for subjects at the same stage, cannot be used in the context of CJD Young et al. (2005).

Based on the clinical assumption that prion disease is highly heterogeneous even among subjects with the same mutation, we chose to extract subject-specific biomarkers. Thus, by extracting subject-specific biomarkers, we ensured that the lack of spatial pattern of biomarkers does not compromise the extraction of features that track subtle brain changes. The extracted imaging biomarkers (Section 3.1) have shown significant differences between healthy controls and symptomatic subjects, for both IPD and sCJD. Nonetheless, the intensity based features, computed from FLAIR images, did not show statistical relevance to separate symptomatic subjects from healthy controls. Currently, only MRI features are considered. For a better accuracy in prediction of prion disease severity or onset, we intend to include quantitative features from other sources, such as blood and CSF biomarkers.

The biomarkers were then used in a non-parametric Bayesian approach to predict the subjects status. The predictive labels are based on the joint modelling of the biomarkers pattern by a Gaussian Process, producing a probabilistic labelling for each subject. We diagnosed independently the sCJD and IPD, evaluating the predictive accuracy of the labels for both subtypes. The reported results, detailed in Section 3.2, are indicative of the effectiveness of the model to detect prion disease patients, among healthy controls. Furthermore, the model was also able to diagnose subjects in the early stages of CJD, particularly for IPD symptomatic subjects with MRC Scale of 20, at which time the diagnosis can be otherwise very challenging. The results also suggest that the diagnosis can be achieved without all three MRI sequences; i.e., the biomarkers extracted from DWI scans and the jointly modelling of FLAIR, DWI and the rate of disease progression are equally predictive for sCJD and IPD when compared with the joint modelling of the three MRI images.

By comparing the predictive accuracy of our model with more simple frameworks, such as SE-SVM, we showed that our model is able to identify symptomatic prion disease subjects with lower uncertainty. Therefore, our model is more relevant in clinical context.

This analysis could yield to improvement of the clinical workflow, since it provides information about which imaging biomarkers are more useful for the earlier diagnosis of CJD and thus avoiding unnecessary exams.

Notwithstanding the promising results obtained for subjects’ diagnosis, there is not an effective characterisation of the different stages of the disease, neither the prediction of clinical onset for IPD patients. This can in part be due to the fact that our model only takes into consideration cross-sectional data and a reduced number of subjects who exhibit high heterogeneity of symptoms.

To improve our knowledge about the evolution of the disease over time, we extended the initial model to perform subject’s staging according to the MRC Scale and by consequence the severity of brain changes. The new model can be seen as a disease progression model, defined as an additive multi-class GP. Contrary to other disease progression models currently used for other neurodegenerative diseases (Lorenzi, Filippone, Frisoni, Alexander, Ourselin, 2017, Oxtoby, Young, Cash, Benzinger, Fagan, Morris, Bateman, Fox, Schott, Alexander), our model does not assume a known ordering of events to stage the subjects in specific clinical status, neither an expected time-to-onset based on the familial clinical onset. Alternately, our model finds the correlation between subjects at the similar stage of the disease, by means of the covariance kernel function. The predicted stages of the disease are then computed based on the highest probability across classes. The overall accuracy (Average Acc = 85%), suggests that the model has been successful in stratifying the subjects based on their MRC Scale score. However, the analysis of the confusion matrix (Fig. 6) suggests that the model is not sensitive to classes with close intervals of the MRC Scale values. The creation of well defined clinical stages of CJD goes beyond the scope of this work, but a future study should investigate alternatives to MRC Scale to define the labels used to train the model for subject’s staging. Furthermore, the current definition of CO is based on the functional disability (MRC Scale equal to 20) and the defined window of one year from the clinical onset; attending to the fast rate of progression of CJD and its diverse evolution among patients, we would need to investigate the best criteria to define this class. Similarly, Asymp. IPD are a very heterogeneous group that should also be stratified itself, in order to have a more accurate and sensitive training of the model.

Thanks to the probabilistic nature of the predictions, the model gives information regarding the predicted class for a given time-point, but also the closest classes for that time-point. This information can be used as a prognosis tool, since the transition between classes can be used to infer the severity of symptoms and consequently the stage of the disease. Bearing this in mind, we intend to test the model for the prediction of the several stages of the disease for the subjects with scans before and after the clinical onset. Only four subjects had all three scans modalities available before and after onset, therefore the results are inconclusive. Moreover, considering the current design of the model, different time-points for these subjects would be modelled independently, which consists in to a source of bias in the model since there is no dependency between results for a same subject. In the future, we will include the subjects excluded from this study due to missing modalities, increasing the sample size and guaranteeing more robust and meaningful results.

Lastly, we also investigated the possibility of using the model as a differential diagnosis tool. The current framework has proven to be able to recognize the individual features of prion disease among another form of dementia: young onset Alzheimer’s disease. Note however that the results reported in Section 3.4 are achieved without a particular modelling for the two types of dementia. The current formulation of the model relies only on the proximity of features pattern for the subjects with the illness. In the future, we plan to adapt the model to learn what is the best covariance kernel function for different neurodegenerative diseases. Following the good results obtained in this illustrative example, we intent to develop a new diagnostic tool based on quantitative measures, which should account for the uncertainty of the diagnosis, given the similarity of prion diseases to other syndromes. This new diagnostic algorithm, developed to identify prion disease among other neurodegenerative diseases, would improve the detection accuracy of this illness, and thus address the current high rate of misdiagnosis patients.^6^

By analysing the predictive accuracy of the latent models, our approach also gives information regarding the combination of input features that better describes the response variable, defined here as subject’s status. This means that we can extend our approach to learn the best model for a specific aim, aside of learning the best kernel function used to explain the variance of the features that characterise the subjects’ symptoms.

More generally, due to their statistical nature, the performances of machine learning approaches are negatively impacted by small sample size in the presence of normal or pathological variability. Finally, a specific drawback of GP models is that their computational complexity scale poorly with the number of observations *N*; i.e., solving GP models requires $O(N^{3})$ computations.

In the future, thanks to the flexibility given by the GP, we aim to extend our framework to account for the longitudinal information available. This will allow not only a more accurate stratification of subjects based on the extracted biomarkers, but also the subjects prognosis in a given time frame. We intend to do this by integrating a spatio-temporal covariance model, such as the Kronecker form proposed by Lorenzi et al. (2015), to provide a unified framework to model jointly the time-series of biomarkers measurements with different natures, for a given subject.

## 5 Conclusion

This study presents a novel framework to extract and select imaging biomarkers especially relevant for the diagnosis of prion diseases. We demonstrate that it is possible to use a non-parametric Bayesian algorithm for the diagnosis and subjects’ stratification by disease severity. Moreover, the model presented in this study may also be extended to consider longitudinal data and to model individual brain changes; therefore, the model represents a promising tool for subjects’ diagnosis and prognosis.

## Declaration of Competing Interest

The authors declare that they have no known competing financial interests or personal relationships that could have appeared to influence the work reported in this paper.

## Footnotes

1 According to clinical experience, the slow progression is seen for A117V, P102L, Y163X, 5– and 6–OPRI, whereas E200K, D178N, E196K and sporadic CJD evolve fast.

2 The * notation refers to the inference for a new sample, using the optimised model.

3 The asymptomatic subjects considered as part of clinical onset (CO) had have the diagnosis confirmed in later MRI images.

4 GPstuff: Bayesian Modeling with Gaussian Processes, available from https://jmlr.csail.mit.edu/papers/v14/vanhatalo13a.htmlVanhatalo et al. (2013)

5 https://www.nitrc.org/plugins/mwiki/index.php/mricrogl:MainPage .

6 Note that the initial symptoms of the disease are usually mistaken as depression symptoms, and even in later stages of the disease, the symptoms may be seen as dementia symptoms, particularly in the inherited form of CJD in which the progression is slower.
